# Molecular docking analysis of hyperphosphorylated tau protein with compounds derived from Bacopa monnieri and Withania somnifera

**DOI:** 10.6026/97320630017798

**Published:** 2021-09-30

**Authors:** Hrushikesh Dixit, C Selvaa Kumar, Debjani Dasgupta, Nikhil Gadewal

**Affiliations:** 1 School of Biotechnology and Bioinformatics, D.Y.Patil Deemed to be University, CBD Belpaur, Navi Mumbai, Maharashtra, India; 2 Advanced Centre for Treatment, Research and Education in Cancer (ACTREC), Kharghar, Navi Mumbai, India

**Keywords:** Alzheimer's disease, Bacopa monnieri, tau protein, bacoside

## Abstract

Tau protein, the major player in Alzheimer’s disease forms neurofibrillary tangles in elderly people. Bramhi (Baccopa Monniera) is often used as an ayurvedic treatment for Alzheimer's disease. Therefore it is of interest to study the interaction of compounds derived from Baccopa with the Tau protein involved in tangle formation. We show that compounds such as bacopaside II, bacopaside XII, and nicotine showed optimal binding features with the R2 repeat domain of hyperphosphorylated tau protein for further consideration in the context of Alzheimer's disease (AD).

## Background:

Alzheimer's disease (AD) affecting mainly the elderly is associated with the nervous system. Two distinct pathologies of AD include amyloid plaque deposition and neurofibrillary tangles of hyperphosphorylated tau protein [1]. Present treatments are symptoms based which only reduces the effect of the disease. However, disease progression can also be arrested by controlling the formation of extracellular amyloid β (aβ) plaque deposition and neurofibrillary tangle formation [2]. As per the treatment regime, phytochemicals can be targeted against extracellular aβ plaques and intracellular neurofibrillary tangles (NFTs). In particular, tangles are made up of tau protein, which is the structural architecture of mitochondria, chromosomes, and nutrient transportation [3]. They are the potential targets in AD for disease-modifying therapies. Targeting tau protein could reduce the production of αβ and tangles by foiling their accumulation [3].

Tau protein, the major player in AD has eight domains classified into N-terminal domain (N1, N2), proline-rich domain (P1 and P2), and four microtubule-binding domains (MBD). These microtubule-binding domains have four repeat domains (R1: 561-591; R2: 592-622; R3: 623-653; R4: 654-685) [4] (Figure 2 - see PDF). In particular, R2 and R3 repeat domains are associated with higher self-aggregation and filament formation. Most importantly, the R3 repeat is the triggering point for molecular aggregation among the four repeat peptides [5]. Approved allopathic drugs like Galantamine, Donepezil, and Rivastigmine, which are the acetylcholinesterase inhibitors (AChEIs) helps in controlling the symptoms of AD [6-7]. Apart from that, ayurvedic medications have also been considered for treating AD due to their neuroprotective phytochemicals. The crude extract of *B. monnieri* and *W. somnifera* was proven to be effective against neurological disorders [8].  But the significant role of bioactive components of both *B. monnieri* and *W. somnifera* has not been investigated from an in-silico perspective. Basically, W. somnifera is a nootropic agent which enhances cognition and is administered to improve mental health and immunity [9-10]. As per reports, alkaloid extract from the root of W.somnifera calms the central nervous system in various mammals [11]. Regarding *B. monnieri*, this perennial creeper helps to improve cognition and cures various nervous disorders [12,10]. Therefore, it is of interest to document the molecular docking analysis data of hyperphosphorylated tau protein with compounds derived from *B. monnieri* and *W. somnifera*.

## Materials & Methods:

### Conformer generation and docking of compounds

Phytochemicals of *B. monnieri* and *W. somnifera* were downloaded from the PubChem database [13] [Table 1 - see PDF].  Additionally, two clinically approved drugs viz. Galantamine and Rivastigmine were also downloaded and considered as control. In total, eighteen chemical compounds from *B. monnieri* and five from *W. somnifera* were considered for docking. As all the downloaded chemical compounds were in 2D format, a stable conformer of each compound was generated using Biovia Discovery studio [14] based on their overall atoms and rotatable bonds. For receptor, the human tau protein was modelled using I-TASSER and further subjected to phosphorylation (*ptau*) and hyperphosphorylation (*hptau*) using and Vienna PTM 2.0 software in our previous study [15]. All twenty-five compounds were docked within the active site of *ptau* and *hptau* using the CDOCKER tool from Biovia Discovery Studio. The active sites were identified based on the available protein crystal structure report. CDOCKER energy scores were generated for each docking. The conformer with the lowest CDOCKER energy was selected for further analysis.

### ADME and Drug likeliness prediction

ADME (Absorption, Distribution, Metabolism and Elimination) studies of all twenty-three chemical compounds and the control drugs were performed using SWISS-ADME online server (http://www.swissadme.ch/) [16] to predict their pharmacokinetic properties. Basically, this study was intended to understand the different physiochemical properties like oral bioavailability, XLOGP3, TPSA, Log S (ESOL), GI absorption, BBB permeability, and Log Kp (skin permeation) of drug molecules. Drug likeness was studied based on Lipinski's rule of five. Furthermore, these results were confirmed with pkCSM-Biosig (http://biosig.unimelb.edu.au/pkcsm/) [18] and MOLINSPIRATION online software [19] for ADME and drug-likeness respectively. SMILE strings of the chemical compounds were supplied to the server.

## Results and Discussion:

The modelled tau protein was phosphorylated and hyperphosphorylated from in silico perspective and later considered for active site identification based on the available tau crystal structure (PDB ID: 2MZ7) bound to microtubules [20]. Thus, four residues identified from the active site pocket of 2MZ7 were mapped on to R2 (Ser606, Ser610 and Ser622) and R3 (Tyr627) domains. Thus, based on the binding affinity, bacopaside II, XII, and nicotine showed better binding with *hptau* compared to *ptau*. Bacopaside II displayed eight and twelve hydrogen bonds with *ptau* and *hptau* respectively. With bacopaside XII, four and seven hydrogen bonds were observed between *ptau* and *hptau* respectively. Nicotine showed two and a single hydrogen bond with *ptau*  and *hptau* respectively (Table 2 - see PDF). The phytochemicals bacopaside II and XII interacted with the R2 and R3 domain in *ptau*. After hyperphosphorylation, interactions were with R2, proline-rich domain2 and C-terminal domain. Importantly, there were no interactions observed between the phytochemicals and the R3 domain (Table 2 - see PDF). However, nicotine maintained its interaction with R2 domain in *ptau* and *hptau*. Furthermore, the phytochemicals of *W. somnifera* showed stronger binding with the *ptau* compared to the *hptau*. In *ptau*, Anaferine interacted with R2 and the R3 domains, which were further, confined to R1 and R2 domain in *hptau*. In essence, these phytochemicals were able to interact only with the R2 domain and not with the R3 domain after hyperphosphorylation due to the major conformational changes within the repeat domain. The non-availability of the R2 repeat domain after the binding of phytochemicals could avert the fibril formation with the R3 domain. Control drug Cusohygrin showed no hydrogen bonds with *ptau* but preferred R2 domain in *hptau*. Even Isopellenterine could not establish a strong binding with the *hptau* (Table 3 - see PDF) (Table S1 -see PDF).

The phytochemicals Bacoside II and Bacoside XII of *B. monnieri* irrespective of their strong interaction with the *hptau* showed poor flexibility, polarity, and size. However, their Log P values were within the permissible limit. Other derivatives of *B. monnieri* like bacopaside III, IV, V, XI, Bacopasaponin A, B, C, D, G, Bacoside A, Bacoside A1, and Bacoside A3 also followed the same trend. In contrast, the derivatives of *W. somnifera* like Anaferine, Anahygrine, Withanolide, cuseohygrin, and Isopellenterine irrespective of their weak interaction with the *hptau* fared well with respect to their physicochemical space for oral bioavailability, pharmacokinetics, and drug likeliness. To confirm these findings, software like PKCSM and MOLINSPIRATION were taken into consideration. As per the PKCSM report, the Caco2 permeability score of BacosideII and XII were out of range < than 0.9. The intestinal absorption of II and XII was 25.49% and 3.78%, which are less than the cut-off score of 30% for better absorption. Thus, the parameters associated with absorption, distribution, and excretion were out of the permissible limit for bacopaside II and XII.  MOLINSPIRATION report also confirms this through their molecular weight, Hydrogen bond donor-acceptor, and drug-likeness (Lipinski rule) (Table 4 -see PDF). As per the ADME study, only nicotine showed permissible values.  Irrespective of favourable binding energy and the pharmacokinetics report, nicotine-based treatment needs to be taken with caution due to their significant role in enhancing tau phosphorylation [23-24]. As recorded by earlier studies, phytochemicals like bacopaside II, XII showed higher flexibility, size, and polarity [25]. Researchers have attempted to enhance the bioavailability and the water solubility of these phytochemicals by loading them into biodegradable nanoparticles [26]. These nanoparticle conversions have already proceeded to enhance the neuroprotective activities of B.monnieri, which assist in improving their therapeutic potential, efficacy, and their specificity [22]. Poly (lactic-co-glycolic acid) PLGA nanoparticle-based delivery of bacoside A and Platinum nanoparticles using B.monnieri (BME-PtNPS) are underway to treat Alzheimer's disease [27].

## Conclusion:

We show that compounds such as bacopaside II, bacopaside XII, and nicotine showed optimal binding features with the R2 repeat domain of hyperphosphorylated tau protein for further consideration in the context of Alzheimer's disease (AD).
